# New Perspectives on Desensitization in the Current Era - An Overview

**DOI:** 10.3389/fimmu.2021.696467

**Published:** 2021-07-30

**Authors:** Vineeta Kumar, Jayme E. Locke

**Affiliations:** Comprehensive Transplant Institute, University of Alabama at Birmingham, Birmingham, AL, United States

**Keywords:** kidney, desensitization, kidney paired donation, novel therapeutic agents, allocation

## Abstract

Blood group and tissue incompatibilities remain significant barriers to achieving transplantation. Although no patient should be labeled “un-transplantable” due to blood group or tissue incompatibility, all candidates should be provided with individualized and realistic counseling regarding their anticipated wait times for deceased donor or kidney paired donation matching, with early referral to expert centers for desensitization when needed. Vital is the careful selection of patients whose health status is such that desensitizing treatment is less likely to cause serious harm and whose anti-HLA antibody status is such that treatment is likely to accomplish the goal of increasing organ offers with an acceptable final crossmatch. Exciting new developments have re-energized the interest and scope of desensitization in the times ahead.

## Introduction

For patients with end-stage renal disease (ESRD), kidney transplantation is the optimal therapeutic option associated with a higher quality of life and lower mortality than chronic hemodialysis. The best results are achieved with a kidney from a healthy ABO and HLA matched living donor. Living donation accounts for a third of all kidney transplants performed in the United States. Unfortunately, not every donor recipient pair is feasible because of human leukocyte antigen (HLA) sensitization or ABO incompatibility (ABOi). To overcome these barriers to transplant, strategies such as desensitization have been developed. Here in we discuss historical and current perspectives on desensitization and reflect on future directions.

## Desensitization: The Historical Perspective

Sensitization to HLA is observed in 30% of patients on the kidney-transplant waiting list and an additional 11% of donor recipient pairs are ABO incompatible (1). This relegates a significant number of kidney transplant candidates with otherwise suitable living donors to the deceased-donor waiting list because of preformed antibodies to HLA and ABO blood group antigens. Desensitization is the removal of circulating donor specific antibodies to HLA or ABO antigens to prevent graft rejection. Crossing HLA and ABO barriers became an emerging phenomenon starting in the mid 90’s and gained significant traction over the next two decades. Use of preconditioning, either with high-dose intravenous immune globulin (IVIg) or with plasmapheresis plus low-dose IVIg, increased transplantation rates, reduced waiting time and had promising short-term outcomes across many single center studies. A survival advantage was established for patients undergoing desensitization for HLA incompatibility followed by live donor transplantation compared with those waiting for a compatible transplant (2). Similarly, candidates who received an ABOi LDKT had higher cumulative survival at 5 and 10 years (90.0% and 75.4%, respectively) than similar patients who remained on the waitlist or received an ABOc LDKT or ABOc DDKT (81.9% and 68.4%, respectively*) (* 3 *).* However, many of these data were collected before implementation of the Kidney Allocation System (KAS), and patients in the control group were not necessarily enrolled in paired exchange programs. Additionally, regardless of the treatment strategy, highly sensitized patients whose calculated panel reactive antibody (cPRA) is ≥98% remained difficult to transplant as desensitization proved to be challenging in those with high HLA or ABO antibody titers.

## Desensitization – The Present Era

Enthusiasm (and need) for desensitization of highly sensitized patients has decreased in recent years. Desensitizing treatments are expensive, resource intensive, and place patients at risk for morbidity associated with higher doses of maintenance immunosuppression. Furthermore, these treatments remove circulating antibody or temporarily inhibit antibody production without significant effect on immunologic memory. Additionally, the survival data are not replicable. The 5-year data from the Mayo Clinic showed significantly worse patient and graft survival in those undergoing desensitization with plasmapheresis and low-dose intravenous immunoglobulin (PP/IVIg) versus HLA compatible transplant, as well as protocol biopsy-detected transplant glomerulopathy in 55% of desensitized versus 7% of HLA-compatible recipients (4). In addition, adjunct therapies such as bortezomib, a potent, reversible proteasome inhibitor that targets terminally differentiated plasma cells, has had limited use secondary to unclear efficacy and adverse side effect profile. The largest study of bortezomib-based desensitization therapy along with plasmapheresis and Rituximab was a prospective trial of 44 sensitized patients conducted in five phases, differing in bortezomib dosing density and plasmapheresis timing, and showed a substantial reduction in immune-dominant anti-HLA DSA. Nineteen of 44 (43%) patients were transplanted during the study period with low acute rejection rates (18.8%) and *de novo* DSA formation (12.5%), demonstrating proteasome inhibitor-based desensitization consistently and durably reduced HLA Ab levels and may be a reasonable alternative to IVIg based plasmapheresis regimens in a select population (5). However, bortezomib as monotherapy in a study of 10 highly sensitized kidney transplant candidates with DSAs against their intended living donor resulted in only a modest reduction in DSAs with no change in CPRA despite use of 32 doses of bortezomib. Not surprisingly, the treatment was not well tolerated due to adverse effects (6). Given these data, the best option would be to avoid HLA-incompatible transplant whenever possible, although not necessarily at the expense of significantly prolonged dialysis exposure while awaiting a compatible offer. Several options in the present era, to include changes in the 2014 KAS for highly sensitized recipients and allocation of A2/A2B donor kidneys to B recipients and kidney paired donation (KPD) allowed for alternative choices for these patients.

KAS prioritizes organ offers for patients with high levels of anti-HLA sensitization providing sensitized patients with a cPRA of 20% and above additional points toward organ allocation priority, and highly sensitized candidates with a cPRA of 100%, 99%, and 98% provided 202.1, 50.09, and 24.4 additional points for allocation priority, with each point being equivalent to 1 year of wait-time (7). In addition, candidates with cPRA of 100% receive priority for kidneys shared nationally. These innovations have reduced the median waiting time for highly sensitized patients with cPRA of 98%–100% from >19 years to 3.2 on the deceased donor waiting list (8). These priority allocation points continue with the new allocation policy that went into effect March 14, 2021.

In the United States, the allocation of deceased donor kidneys is based on ABO matching as opposed to ABO compatibility. The waiting time for blood type B kidney transplant candidates is typically longer than for candidates with other blood types. The blood type B kidney transplant candidate waitlist has the highest proportion of ethno-racial minority candidates, who are less likely to receive a living donor kidney transplant compared to white candidates, exacerbating longer waiting times for patients in these populations. With the goal of improving equity by improving access to transplantation for minority populations in the United States, KAS now allows allocation of type A, non-A1 and type AB, non-A1B (commonly known as A2 and A2B) kidneys to blood type B candidates if anti-A titers are low, with the program’s titer threshold defined in written policy.

Finally, KPD or paired kidney exchange has become increasingly utilized as an approach to overcome biologic incompatibility, wherein two or more incompatible donor-recipient pairs exchange kidneys and all recipients benefit by receiving compatible transplants. The scope of KPD has further expanded with introduction of non-directed donors and compatible pairs in the form of multi-way exchanges and kidney donor chains to name a few. This has allowed the practice of KPD in the United States to expand exponentially, from a handful of transplants per year when it first started in 2002 to over 1000 transplants in 2020. An additional novel approach has become the combination of both KPD and desensitization to facilitate compatible and successful transplantation. An HLA sensitized patient pair can be paired with a better immunological match in the KPD pool than the original donor and subsequently desensitized to achieve transplantation establishing KPD and desensitization are not mutually exclusive strategies. Importantly, the development and implementation of KPD programs has been demonstrated to mitigate racial and gender disparities in access to living donor kidney transplantation (9).

While the above options have reduced the need for desensitization, it has not been made redundant. Despite KAS, the most highly sensitized >99.5% PRA candidates on the wait list still do not get their fair share of transplants. Only 9.7% (213/2204) of candidates with a calculated panel reactive antibody ≥99.9% received a transplant, and the most highly sensitized candidates were less likely to receive a living donor transplant. Among candidates with a CPRA ≥ 99.5% (i.e. 100%), only 2.5% of transplants were from living donors (13 total, 7 from KPD). Nearly 4 years after KAS (6/30/2018), 1791 actively wait-listed candidates had a CPRA of ≥99.9% and 34.6% (620/1791) of these had ≥5 years of waiting time (10). Thus, despite KPD and KAS, the most highly sensitized candidates have not been transplanted even with prolonged waiting time. Candidates with a CPRA ≥ 99.9% and sensitized candidates with an incompatible living donor and prolonged waiting time may benefit from desensitization to improve their ability to receive a transplant.

Despite the changes in KAS for Blood type B recipients, there is still a vast number of Blood type O and B wait list candidates with disproportionately longer wait times facing a high mortality while they wait. Clinical outcomes after ABO antibody reduction in ABO-incompatible living-donor kidney transplant recipients are not much different from those achieved in ABO-compatible control groups and desensitization for ABOi remains an unmet need (11).

## Desensitization: Future Directions

Exciting developments in the field of immunosuppression, diagnostics and therapeutic options continue to offer hope to the subset of patients that are considered “un-transplantable” *via* desensitization with and without KPD.

- Imlifidase is a promising new Investigational therapeutic. The IgG-degrading enzyme derived from *Streptococcus pyogenes* (Imlifidase; IdeS) is a recombinant cysteine protease that cleaves all four subclasses of human IgG into F(ab’)2 and Fc fragments, inhibiting CDC and antibody-dependent cellular cytotoxicity. The efficacy of Imlifidase as a desensitization strategy was evaluated in two independent phase I/II trials (12). Treatment with Imlifidase produced complete cleavage of IgG into F(ab’)2 and Fc fragments within six hours of infusion. Intact IgG remained absent in all patients for at least seven days, and there was a persistent reduction in IgG levels at 28 days after infusion. IVIG and rituximab following Imlifidase was associated with less donor specific antibody (DSA) rebound, with the median fluorescent intensity (MFI) of the immunodominant DSA reported to be 0 at one month. Mean estimated GFR (eGFR) at one to six months posttransplant was 70 mL/min/1.73 m2 in the United States study.
- Obinutuzumab is a third-generation anti-CD20 monoclonal antibody less reliant on complement-dependent cytotoxicity and is mediated primarily through antibody-dependent cellular cytotoxicity with effective B cell depletion not only in the peripheral blood but also in the secondary lymphoid organs and may have more lasting effects on memory B cells and plasma cells than Rituximab.
- Carfilzomib is a second-generation, irreversible proteasome inhibitor that has a more favorable toxicity profile than bortezomib with a pronounced reduction in DSA MFI when used in combination with plasma exchange in a small study of 12 highly sensitized patients (13).
- Tocilizumab is a monoclonal antibody directed against the IL-6 receptor that is being used for treatment of rheumatoid arthritis. In one pilot trial of ten transplant candidates who had failed desensitization with IVIG and Rituximab and were subsequently treated with IVIG and tocilizumab, five were successfully transplanted (14).

An Achilles heel in ABOi transplantation is the variable correlation of traditional anti-A and Anti-B blood group titers detected *via* hemagglutination with graft loss from acute antibody mediated rejection early post-transplantation. In contrast to the hemagglutination assay, the ABO-glycan microarray allows detailed characterization of donor-specific antibodies necessary for effective transplant management, representing a major step forward in precise ABO antibody detection *(* 15 *).* Characterization of ABH antigen subtype expression in other organs such as kidney and liver will be valuable for its wider application in ABOi organ transplantation. The glycan microarray may allow reliable assessment of patients for their suitability to receive an ABOi transplant and for appropriate pre-and posttransplant clinical management. Furthermore, by accurately assessing the absence of antibodies specific for graft antigens, unnecessary interventions may be avoided such as antibody removal by plasmapheresis or aggressive immunosuppressive therapies.

An exciting application to this technology is the development underway of silica microparticles functionalized with A and B blood group carbohydrate antigens (A type I, A type II, B type I, and B type II) to enable the detection and monitoring of ABO antigen-specific B cells much like the single antigen bead analysis in HLA testing. This approach therefore comprises a novel, general platform for screening B cell populations for binding to carbohydrate antigens, including, in this case, the human A and B blood group antigens (16).

Patients with DSA and T-cell activation as demonstrated by high levels of soluble CD30 (sCD30) in pretransplant serum have a threefold higher risk of graft loss than patients with DSA but low sCD30 levels *(* 17 *).* Using this and other novel biomarkers to follow treatment response in addition to traditional DSA MFI/titer measurement may offer additional guidance into management before and after transplantation. Furthermore, the conventional HLA mismatch has been challenged recently by the concept of HLA epitope matching algorithms that claim to offer a more precise assessment donor recipient HLA compatibility. Molecular mismatch has been proposed as a prognostic biomarker categorizing individual donor recipient pairs into low and high risk where for every 10 eplet mismatches there is a 2-fold increased hazard of developing a DR or DQ antibody (18). When individuals were categorized based on thresholds of eplet mismatch into low or high risk there was a prognostic co-relation for both cellular and antibody mediated rejection (19). Furthermore, patients at low HLA DR/DQ mismatch were able to tolerate less immunosuppression and still had less rejection and *de novo* DSA formation (18). This is promising for future adoption in the field of desensitization where one could better define acceptable mismatches *via* epitope matching and also try and cross low level epitope mismatches more safely while avoiding the high-risk phenotypes. Rate limiting factors for current adoption include lack of universal high-resolution typing for every donor and recipient pairs and time consuming and labor intensive testing that is often impractical especially in allocation schemes that have a 12 -24 hr turnaround time. HLA epitope matching, however, has a promising role in living donor kidney transplantation and KPD matching.

In non-kidney solid organs transplant candidates with a high wait list mortality where dialysis and living donors is not an option, desensitization for deceased organ donor transplants continues to have a role and lessons learned in desensitization in kidney transplantation paves the way for future innovations.

## Conclusions

When living donors are available, paired exchange should be attempted to avoid the cost and risk associated with desensitizing therapy as well as the posttransplant immune response that will likely translate into worse long-term graft survival. Paired exchange options should be exhausted, and a realistic estimate of wait time taking into account priorities for high cPRA patients under KAS should be considered. Proceeding with desensitization for those highly sensitized patients without living donors, where paired exchange is not possible and expected wait time is considered unacceptable, may be a reasonable consideration. Although no patient should be labeled “un-transplantable” due to blood group type or DSA, all candidates should be provided with individualized and realistic counseling regarding their anticipated wait times for deceased donor or kidney paired donation matching, with early referral to expert centers when needed. Careful patient selection, which involves the identification of individuals who can withstand desensitization treatment and have favorable antibody profiles amenable to successfully overcoming the incompatibility to allow transplantation, remains the cornerstone desensitization. One cannot emphasize enough the importance of careful antibody monitoring throughout the posttransplant period. Lessons learned from the past along with exciting developments in pipeline will pave the way for the future of desensitization.

## Author Contributions

All authors contributed to the article and approved the submitted version.

## Conflict of Interest

JL: consultant for Hansa (manufactures imfilidase).

The remaining author declares that the research was conducted in the absence of any commercial or financial relationships that could be construed as a potential conflict of interest.

## Publisher’s Note

All claims expressed in this article are solely those of the authors and do not necessarily represent those of their affiliated organizations, or those of the publisher, the editors and the reviewers. Any product that may be evaluated in this article, or claim that may be made by its manufacturer, is not guaranteed or endorsed by the publisher.
